# Classifying Learning Speed Using Brain Networks and Psychological States: Unraveling the Interdependence Between Learning Performance, Psychological States, and Brain Functions

**DOI:** 10.7759/cureus.70133

**Published:** 2024-09-24

**Authors:** Hiroki Bizen, Daisuke Kimura

**Affiliations:** 1 Department of Occupational Therapy, Faculty of Health Sciences, Kansai University of Health Sciences, Osaka, JPN; 2 Department of Occupational Therapy, Faculty of Medical Sciences, Nagoya Women’s University, Nagoya, JPN

**Keywords:** brain network, near-infrared spectroscopy, performance learning, psychological state, rehabilitation

## Abstract

Introduction

The progression of performance learning (PL) may have complex relationships beyond mere concurrent occurrences and may influence each other. This study aimed to classify the speed of PL using a random forest based on brain network and stress state information and to identify the factors necessary for PL. In addition, this study also aimed to clarify the complex interdependent relationships between PL, psychological state, and brain function through these factors, using covariance structure analysis.

Methods

A total of 20 healthy individuals participated in a choice reaction time task, and brain function was measured using near-infrared spectroscopy (NIRS). Participants were divided into high-PL and low-PL groups based on the median difference in correct responses.

Results

Random forest analysis identified the left orbitofrontal area, right premotor cortex, right frontal pole, left frontal pole, left dorsolateral prefrontal cortex, and depression and anxiety as key factors. Covariance structure analysis revealed that depression and anxiety affected PL through the frontal pole and prefrontal cortex, suggesting a complex interplay between psychological state, brain function, and learning.

Conclusions

These findings suggest that psychological states influence brain networks, thereby affecting learning performance. Tailoring rehabilitation programs to address psychological states and providing targeted feedback may improve learning outcomes. The study provides insights into the theoretical and practical applications of understanding the brain's role in PL, as well as the importance of addressing psychological factors to optimize learning and rehabilitation strategies.

## Introduction

Performance learning (PL) is affected by several factors, including motivation, feedback, and stress [1-4]. From the perspective of brain function, PL is an outcome of changes in the brain [5,6]. As PL progresses, the brain forms networks between regions and undergoes changes [7,8]. Understanding the roles of, and changes in, brain regions during PL is important for optimizing interventions to improve performance and creating tailored strategies based on objective brain function. Furthermore, post-stroke patients often experience stressful conditions, such as anger, helplessness, apathy, and fatigue [9,10]. While moderate stress can positively affect learning and performance, excessive stress can have negative effects [3,4]. Additionally, mood influences learning and performance [11]. Thus, various factors could intricately influence each other during PL, potentially leading to skill acquisition.

Increasing research on brain function analysis has aimed to elucidate brain networks based on graph theory. Furthermore, studies have considered regions with correlated brain activity as functionally connected, despite the anatomical distance. Research has also employed various indices, such as betweenness centrality, characteristic path length, and clustering coefficients [12]. Among these, regions with high betweenness centrality serve as important hubs in complex networks and aid in the evaluation of the efficiency and speed of information transmission in brain networks [13]. Therefore, this study focused on betweenness centrality.

Recently, machine learning has made advancements in predicting classification models. Random forest, a machine learning algorithm, is effective in suppressing overfitting by constructing multiple decision trees via bootstrap methods. Furthermore, it analyzes the importance of each feature in the classification.

Psychological states influence both the mind and body, which can lead to various bodily reactions [14]. Previous studies suggested that psychological states affect brain function. Furthermore, the dorsolateral prefrontal cortex, in particular, plays a significant role in novel learning [15]. Therefore, these elements could have complex relationships beyond mere simultaneous occurrences and could mutually influence each other.

This study aimed to classify the speed of PL via random forest based on brain network and stress state information and to identify the factors necessary for PL. Additionally, we also aimed to clarify the complex interdependent relationships between PL, psychological state, and brain function through these factors.

If learning speed could be classified based on brain function and psychological state, early individualized rehabilitation programs could be provided. Additionally, understanding the influence of psychological states on PL can provide valuable insights for clinical research. If psychological states influence PL via brain function, they could serve as a basis for implementing multidimensional rehabilitation approaches. Furthermore, the results can provide insights regarding both theoretical aspects and practical applications.

## Materials and methods

Participants

This study involved 20 healthy individuals (10 males, 10 females), with a mean age of 26.5 ± 5.0 years. The inclusion criterion was participants with a confirmation of right-handedness via the Edinburgh Handedness Inventory [16]. The exclusion criterion was those with a history of organic brain disorders, such as stroke. Participation was voluntary. The study was explained to them verbally and in writing, and they were made aware that participation was voluntary and that they could withdraw at any time. The study was conducted with approval from the Ethics Committee of the lead author's institution (Approval No. 21-14).

Learning task

**Figure 1:**
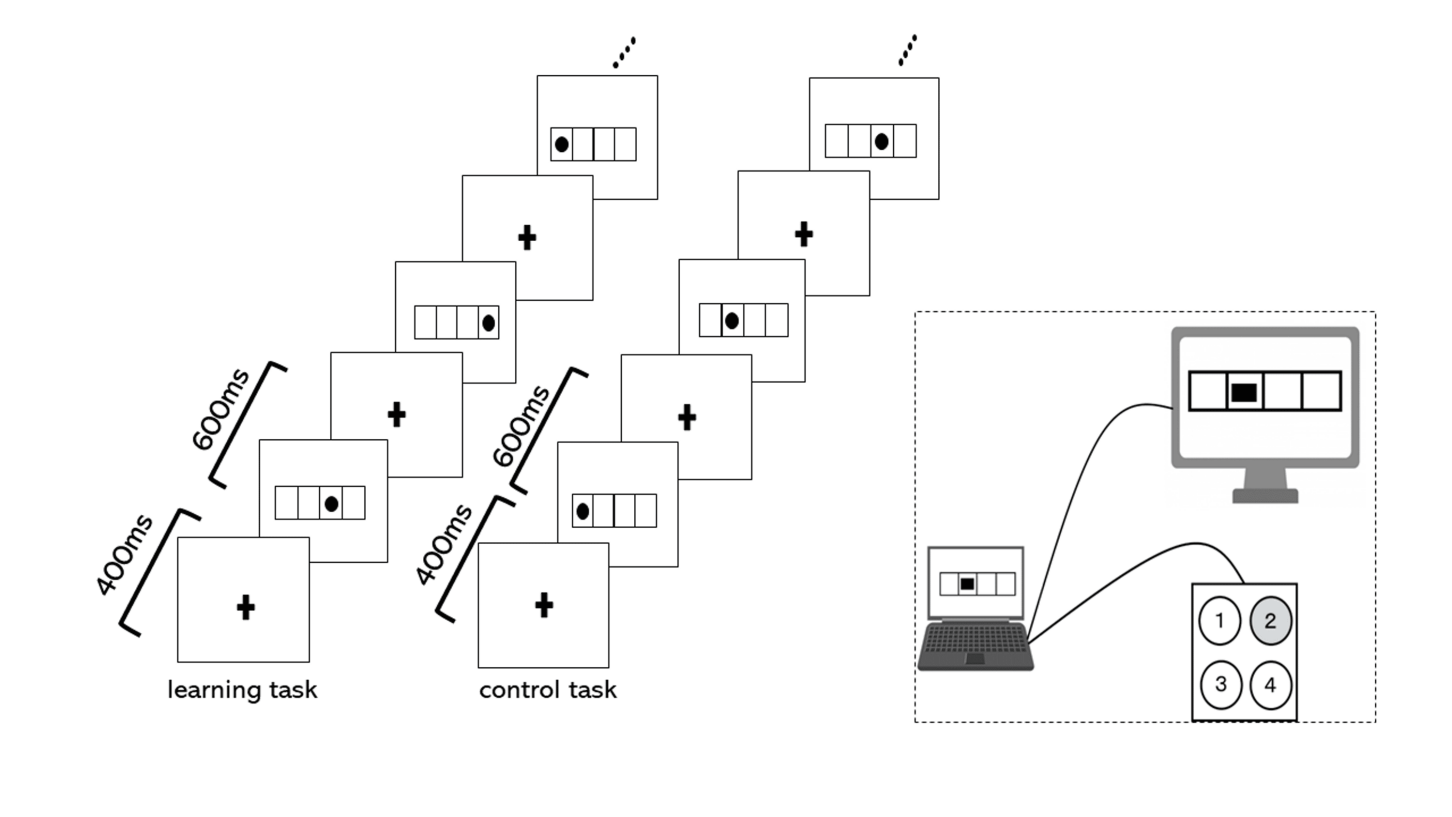
Learning task Participants were required to press a button that corresponded to the location of a lit symbol within one of four frames labeled 1-4 from left to right. We performed a control task in which symbols were regularly illuminated in the sequence of 1-2-3-4 to eliminate brain activity associated with simple movements other than the learning task. The learning and control tasks lasted 20 and 30 seconds, respectively. Each task was repeated three times alternately.

Figure 1 illustrates the choice reaction time task. Participants were required to press a button that corresponded to the location of a lit symbol within one of four frames labeled 1-4 from left to right. Performance was measured by the number of accurate button presses, referred to as “correct responses.” The order of symbol illuminations was randomized. Symbols were illuminated for 600 ms, followed by a 400 ms presentation of a plus symbol at the center between illuminations.

Experimental protocol

The experimental protocol comprised an initial execution as a pre-learning task, a five-minute practice session, and a post-learning task, identical to the pre-learning task.

Near-infrared spectroscopy (NIRS) measurement method

Brain function was measured via NIRS (Hitachi OT-R41; Hitachi, Tokyo, Japan). The sampling frequency was set to 10 Hz, and changes in oxygenated hemoglobin (Oxy-Hb) concentration were recorded. Participants were instructed to maintain a relaxed seated position and hold a button placed on their right thigh with their right hand, while their trunk was supported by the backrest. They were also instructed to avoid body movements and speaking during the NIRS measurement.

Probes were positioned based on the International 10-20 system. A 3 x 11 holder was centered on Fpz and covered the regions of interest, which included the bilateral dorsolateral, prefrontal, and premotor cortexes; inferior frontal; superior temporal; and middle temporal gyrus; orbitofrontal area; and frontal and temporal poles.

Virtual registration was used to align the channel positions with the brain regions. This method projected the position of the virtual probe holders onto the Montreal Neurological Institute standard brain coordinate system, allowing for the estimation of the brain regions measured via the channels, with a spatial estimation accuracy of 13 mm [17].

Furthermore, we performed a control task where symbols were regularly illuminated in the sequence of 1-2-3-4 to eliminate brain activity associated with simple movements other than the learning task. The learning and control tasks lasted 20 and 30 seconds, respectively. Each task was repeated three times alternately.

Network analysis

Networks were represented as nodes and edges. Conversely, brain networks were represented by regions of interest as nodes and functional connections as edges [14]. We defined edges as connections between the regions, with Pearson's correlation coefficients above a certain threshold. These were based on the time-series data of 16 regions of interest obtained from the NIRS analysis [18,19].

Correlation matrices were created via the correlation coefficients between the regions for the 20-second (200-point) time-series data. Matrices were binarized at thresholds that ranged from the top 10% to 30% of the correlation coefficients, with increments of 1%. Network analysis was performed based on graph theory. Furthermore, the average betweenness and centrality of each region of interest were calculated for all the thresholds [18]. GRETNA (MATLAB R2020a) was used for network analysis [20,21].

Stress state evaluation

Stress state was assessed via a psychological stress reaction scale with established reliability and validity. This scale comprised three factors (depression/anxiety, moodiness/anger, lethargy), rated on 18 items that measured various psychological stress reactions experienced in daily life [22].

Mood state evaluation

Participants' mood states were assessed via a two-dimensional mood scale that comprised eight items and allowed for self-monitoring of psychological states regarding vitality, stability, pleasure, and arousal. Previous studies have confirmed its reliability and validity. Feelings of “calm,” “irritated,” “apathy,” “energetic,” “relaxed,” “tense,” “lethargy,” and “lively” were rated on a six-point Likert scale that ranged from 0 (not at all) to 5 (extremely) [23].

In addition, participants rated their level of fatigue and motivation on a five-point scale administered immediately before the experiment.

Statistical analysis

Participants were divided into two groups via the median split method: the high-PL and low-PL groups. This method was chosen owing to the moderate sample size, which minimized the influence of outliers and was widely used in previous studies [24]. We calculated the difference between the number of correct responses in the post- and pre-learning tasks. Furthermore, we calculated the median difference in correct responses and assigned participants with differences less than or more than the median to the high-PL and low-PL groups, respectively.

Sex distributions between the two groups were compared via Chi-squared tests. Age, changes in the number of correct responses, and betweenness centrality of each region were compared via a Mann-Whitney U test.

A classification algorithm was developed via random forest to classify the two groups and extract the important factors. The algorithm used the betweenness centrality of each region of interest in the pre-learning task, sub-items of the stress response scale, two-dimensional mood scale, and subjective fatigue and motivation as learning data. Data were divided into five folds for the five-fold cross-validation. Each fold was randomly assigned as either training or test data. Training data were used to train the algorithm. Subsequently, it was used to predict the classification of the test data. The accuracy of the training and testing data for each fold was calculated and averaged across the five folds to evaluate classification accuracy. The mean decrease in the Gini coefficient, a significant classification index, was calculated for each fold and averaged across the five folds. “RandomForest,” a package of R version 4.3.0 (R Foundation for Statistical Computing, Vienna, Austria), was used for data analysis.

In addition, the mean decrease in Gini values was sorted in descending order, and cumulative contribution rates were calculated. A confirmatory factor analysis (CFA) was performed on the top items that exceeded 50% to create a hypothesized model, which suggested that the separated factors influenced learning. Subsequently, a structural analysis of covariance was performed. Model fit was assessed via several indices, which included the goodness of fit index (GFI), adjusted GFI (AGFI), Tucker-Lewis index (TLI), comparative fit index (CFI), and root mean square error of approximation (RMSEA). IBM SPSS Statistics for Windows, Version 23 (released 2015; IBM Corp., Armonk, NY, USA) was used for statistical analysis.

## Results

The median number of correct answers was 6 and 3.5 for the high and low advanced groups, respectively, indicating a significant difference. However, no significant differences were observed for the other items.

The mean of the five-fold accuracy of the training and test data was 100 ± 0% and 41.6 ± 11.8%, respectively.

**Figure 2:**
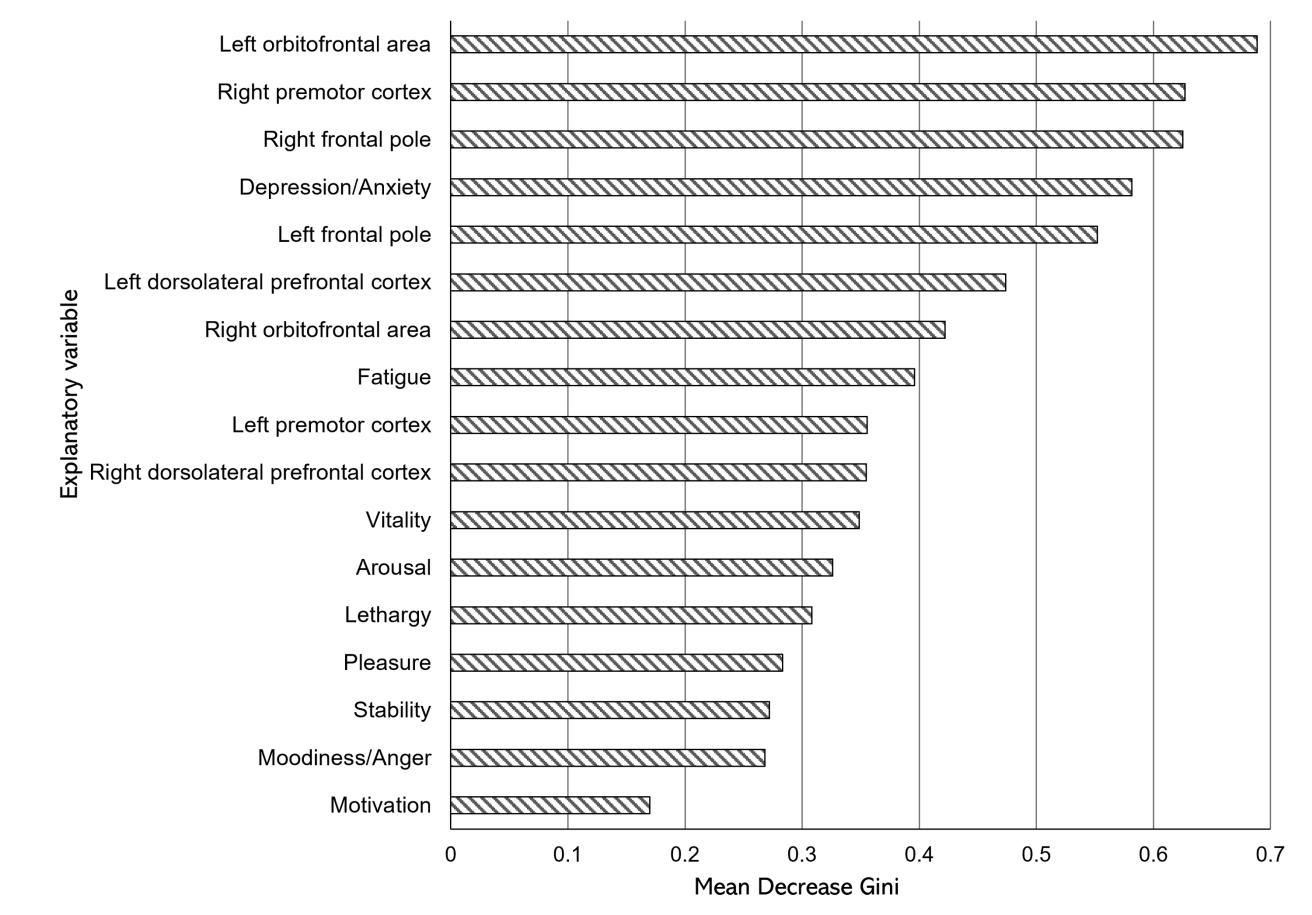
Results of mean decrease in Gini

Areas of interest, in descending order of the mean decrease in Gini coefficient, were the left orbitofrontal area, right premotor cortex, right frontal pole, depression and anxiety, left frontal pole, left dorsolateral prefrontal cortex, right orbitofrontal area, fatigue, left premotor cortex, right dorsolateral prefrontal cortex, vitality, arousal, lethargy, pleasure, stability, moodiness and anger, and motivation (Figure 2).

Subsequently, the cumulative contribution rate of the mean decrease in Gini coefficient exceeded 50% for the top six variables. Therefore, a CFA was conducted for the left orbitofrontal area, right premotor cortex, right frontal pole, depression and anxiety, left frontal pole, and left dorsolateral prefrontal cortex. Consequently, the first factor comprised the right and left frontal poles and was named "frontal pole." The second factor comprised the "right premotor cortex" and "left orbitofrontal area" and was named "prefrontal cortex." The third and fourth factors were "depression/anxiety" and "left dorsolateral prefrontal cortex," respectively.

**Figure 3:**
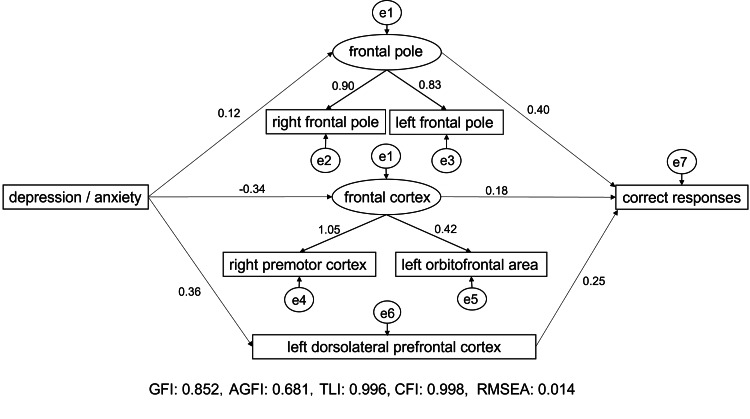
Results of covariance structure analysis GFI: Goodness of fit index; AGFI: Adjusted goodness of fit index; TLI: Tucker-Lewis index; CFI: Comparative fit index; RMSEA: Root mean square error of approximation

The model generated a GFI of 0.852, an AGFI of 0.681, a TLI of 0.996, a CFI of 0.998, and an RMSEA of 0.014, confirming model fit. Latent variables are shown as ovals, observed variables as rectangles, and causal relationships as arrows. The numbers adjacent to the arrows represent the strength of the relationship between the estimated variables; the higher the number (absolute value), the stronger the relationship. CFA results led to the creation of a hypothesized model, which proposed that the "frontal pole" and "frontal cortex" were latent variables. In addition, "depression/anxiety" and "left dorsolateral prefrontal cortex" were observed variables that influenced PL. A covariance structure analysis yielded results of GFI = 0.852, AGFI = 0.681, TLI = 0.996, CFI = 0.998, and RMSEA = 0.014 (Figure 3). Standardized coefficients from "depression/anxiety" to "frontal pole" and "frontal cortex," and from "left dorsolateral prefrontal cortex" to "correct responses" were 0.12, -0.34, and 0.36, respectively. Standardized coefficients from "frontal pole" to "frontal cortex" and "left dorsolateral prefrontal cortex" to "correct responses" were 0.40, 0.18, and 0.25, respectively (Figure 3). It was interpreted that psychological states such as depression and anxiety affect PL via the frontal orbital cortex, left dorsolateral prefrontal cortex, and prefrontal cortex.

## Discussion

The left orbitofrontal area was involved in decision-making and response selection based on reward value [25]. Awareness of performance improvement led to rewards that contributed to the learning progress. Hence, it was connected to the dorsolateral prefrontal cortex. Therefore, we speculated that the left anterior cingulate and dorsolateral prefrontal cortices influenced the pace of PL.

The bilateral frontal pole was identified as one of the top six variables. It acted as a hub to maintain goal-directed persistence and was heavily involved in learning. Previous research demonstrated that the frontal pole played the role of a hub that maintained the sustainability of goal orientation and was deeply involved in learning [26]. Furthermore, it was involved in higher-level executive functions. Hence, individuals were strongly involved in acquisition through practice, without being conscious of the process [27]. Therefore, elements necessary for complex tasks, strategy construction, and PL could depend on the frontal pole.

Conversely, regarding psychological status, "depression/anxiety" also ranked among the top six variables. People with depressive symptoms had reduced blood flow in the frontal pole compared with healthy people [28]. Furthermore, our results indicated that the frontal pole influenced PL on both sides. Therefore, the PL process was hindered due to reduced frontal pole function. Additionally, "anxiety/depression" also impacted slowness in PL. People with high anxiety had less activity in the prefrontal cortex, and additional cognitive resources were used to achieve performance [29]. Therefore, they were less able to adapt to new PL and more likely to exhibit slow PL.

Results of the covariance structure analysis revealed that psychological states, such as depression and anxiety, influenced PL via the frontal pole, left dorsolateral prefrontal cortex, and prefrontal cortex. Therefore, psychological state, brain function, and PL did not interact individually. However, they had a rather complex relationship in which they influenced each other. These results suggested that changes in psychological state altered brain function networks and influenced PL.

Our findings suggest that focusing on basic tasks, closely monitoring PL progress, and setting small, achievable goals to enhance the sense of accomplishment are important for people with slow PL. Reducing anxiety could lead to progress in PL. Conversely, for people with quick learning performance, setting high goals may lead to a sense of accomplishment when performing tasks at a high level and accelerate PL progress. Furthermore, specific feedback on performance reduces anxiety and improves performance [30]. Providing appropriate feedback in parallel with a physical functioning approach reduces anxiety and increases efficiency. This could increase the possibility of obtaining a new performance.

Limitations

This study was conducted on young healthy subjects, and in order to apply this to rehabilitation, it is necessary to continue the study on stroke patients. In addition, since there were 20 subjects in the study, it is necessary to increase the sample size and conduct further studies.

## Conclusions

The pace of PL was influenced by several factors, which included the left orbitofrontal area, right premotor cortex, right frontal pole, depression and anxiety, left frontal pole, and left dorsolateral prefrontal cortex. In addition, the covariance structure analysis revealed complex relationships between PL, psychological states, and brain function. This suggested that psychological states, such as depression and anxiety, affected PL through the brain regions, which included the anterior cingulate, left dorsolateral prefrontal cortex, and prefrontal cortex.
